# Determinants of general health perception among individuals with chronic low back pain overtime: structural equation modeling

**DOI:** 10.1371/journal.pone.0324101

**Published:** 2025-05-23

**Authors:** Owis Eilayyan, Line Enjalbert-Auneau, Sara Ahmed

**Affiliations:** 1 Physical Therapy Department, Faculty of Allied Medical Sciences, Al-Ahliyya Amman University, Amman, Jordan; 2 School of Physical & Occupational Therapy, McGill University, Montreal, Quebec, Canada; 3 Center for Outcomes Research and Evaluation, Research Institute of the McGill University Health Centre, Montreal, Quebec, Canada; 4 Centre de Recherche Interdisciplinaire en Réadaptation (CRIR), Constance Lethbridge Rehabilitation Center, Montreal, Quebec, Canada; Mie University Graduate School of Medicine, JAPAN

## Abstract

**Background:**

Low back pain (LBP) is a public health problem. General health perception is the best predictor of healthcare utilization and mortality. Identifying predictors of health perception helps understand how people with LBP live, implement the appropriate treatment, and improve the quality of care.

**Objective:**

This study aimed to estimate the relationships between pain intensity, psychological distress, self-efficacy, functional ability, and healthcare utilization among individuals with chronic LBP over a period of six-months and to estimate the impact of these relationships on general health perception.

**Methods:**

This is a secondary analysis of data from a longitudinal study that assessed the health outcomes of individuals with chronic LBP. Structural equation modeling (SEM), based on health frameworks, was used to estimate the predictors of health perception among people with LBP at baseline and 6-months.

**Results:**

314 individuals with LBP were included in the analysis. The final SEM model had good fit statistics and explained 48% of health perception variance at 6-months. The model showed that health perception was significantly affected by pain intensity (β = 0.29, β = 0.21), psychological distress (β = 0.51, β = 0.44) and self-efficacy (β = 0.4, β = 0.36) cross-sectionally and overtime, respectively.

**Conclusion:**

Different health outcomes could affect the health perception among people with low back pain. This requires holistic approaches to treatment, involving self-management and cognitive behavioral therapy, as well as improved self-efficacy to improve their health.

## Introduction

Low back pain (LBP) is the most common musculoskeletal condition worldwide [1,2]; approximately 7.5% of the global population suffers from LBP [2]. LBP affects people physically, psychologically, and economically. It is also a major contributing factor to the number of years that a person lives disabled [2]. LBP may lead to problems with physical function, and psychological disorders such as anxiety and depression [3–6]. The literature shows that chronic LBP (CLBP) causes pain interference in activities of daily living and function, poor health-related quality of life (HRQL), and disability [7–11]. An estimated $6–$12 billion is spent on LBP medical care each year in Canada [12].

General health perception (GHP) is considered an important health outcome that can be affected by LBP and other health conditions [13]. It was defined by Wilson and Cleary (1995) as “a subjective overall rating of health”, and it has been shown that GHP can be affected by different health outcomes (e.g., pain, depression, and functional status) [13]. GHP is considered the best predictor of healthcare utilization and mortality [13]. Identifying predictors of GHP helps with understanding the progress of LBP, adjusting treatment to the needs of individuals with LBP, and reinforcing the requirement of multidisciplinary intervention. These may result in optimizing functional ability, GHP, and QoL [14–17]. In addition, studying the GHP indicators and addressing them may prevent the deterioration of patients’ health and enhance self-management approach [18].

The literature shows the factors that significantly enhance GHP in individuals with LBP, CLBP, or chronic pain (including CLBP) include less pain severity, fewer psychological symptoms (i.e., depression and anxiety), higher self-efficacy, and higher functional status [19–32].

However, the studies that were used to conclude the statistical correlations between the different health outcomes relied mainly on cross-sectional design and regression/bivariate statistical approaches. With such approaches, it is not possible to evaluate the interplay between the variables that influence GHP, and it is difficult to indicate the sequence of the factors that influence the disease or the progression of it [33]. Therefore, studying the predictors of GH overtime is important to accurately estimate the effects of different health and social factors on GHP. Furthermore, a more sophisticated analytical approach is needed to incorporate the relationships between predictors of GHP, which may help evaluate the combined effect of these variable on GHP.

The objective of this study was to estimate the relationships between pain intensity, psychological distress, self-efficacy, functional ability, and healthcare utilization among individuals with CLBP over a period of six-months and to estimate the impact of these relationships on GHP.

Guided by Wilson and Cleary and the International Classification of Functioning, Disability, and Health (ICF) models [13, 34, 35], a specific theoretical model was proposed in this study to specify the relationships between GHP and other health outcomes over time in individuals with CLBP.

## Materials and methods

### Population

The study sample included individuals with CLBP who were enrolled in a primary care interdisciplinary LBP program, were 18 years and older, and could speak English and/or French.

### Study Design and Procedure

This study is a secondary analysis using data from a longitudinal study that assessed the health outcomes of individuals with CLBP at baseline and 6-months follow-ups. The original study was conducted to develop and evaluate the impact of interdisciplinary intervention and self-management on different patient outcomes and healthcare utilization over a 2-year period [36]. The program was implemented in four Health and Social Services Centres (CSSSs) in Quebec. The interdisciplinary program included interventions by physicians, nurses, physiotherapists, and psychologists.

The current study’s population was recruited from four interdisciplinary intervention centers from August 1, 2012, to February 20, 2016. The participants completed a package of measurement tools at baseline and at 3 and 6-month follow-up. The nurse case manager provided the patients with the questionnaires during their visits to the clinic. The McGill University Health Center IRB provided ethics approval for the original study (#MP-CUSM-12–220 GEN/2013–999). A written consent form was signed by participants to participate and use their data for research purposes. Participants who only completed questionnaires at both baseline and 6-month follow-up were included in this study. Therefore, the follow-up rate was 100%.

### Measures & Data Collection

All the following measurement tools were completed by participants at baseline and at 6-month follow-up during the clinic visit. Table 1 presents the study’s latent variables and their indicators.

**Table 1:** Latent factors’ indicators and the measurement model.

Variable	Indicators	Factor Loading
**Latent Variable**		
Self-Efficacy	SE1 SE2 SE3 SE4 SE5 SE6 SE7 SE8	0.68 0.47 0.8 0.75 0.8 0.74 0.92 0.83
Pain	BPI-Severity BPI-Interference SF-12 Bodily Pain	0.71 0.71 0.87
Psychological Distress	HADS – Depression HADS – Anxiety PHQ-9	0.92 0.72 0.86
Functional Status	ODI SF-12 Physical Function SF-12 Role Physical SF-12 Social Function	0.88 0.62 0.72 0.92

BPI: Brief Pain Inventory; HADS: Hospital Anxiety and Depression Scale; ODI: Oswestry Disability Index; PHQ: Patient Health Questionnaire; SE: Self-efficacy; SF-12: Short Form 12.

### Outcome: GHP

#### SF-12: General Health Subscale.

The SF-12 is a self-administered short-form questionnaire with 12 items that measure eight dimensions: bodily pain, vitality, mental health, physical functioning, role-physical, role-emotional, social functioning, and general health. The general health item of the SF-12 was used in this study as the outcome. It is rated on a 5-point scale, from poor to excellent [37]. The literature supports the validity and reliability of using a single-item measure [38,39].

### Predictor variables

#### Pain.

Pain was measured by the subscales of the BPI: pain severity and pain interference, and the bodily pain subscale of the SF-12.

##### Brief Pain Inventory (BPI):

Pain intensity and Pain Interference

The BPI is a self-administered tool used to assess pain severity and the impact of the pain on the main aspects of life. BPI has fifteen items and two subscales: pain severity and pain interference. Each item is rated on an eleven-point scale, from 0 to 10, where 0 indicates no problems and 10 indicates maximal problems [40]. The reliability and validity of BPI are supported by the literature [41].

#### SF-12: Bodily Pain Subscale.

The bodily pain subscale of the SF-12 was used in this study as one of the indicators of pain. It includes one item rated on a 5-point scale, from “not at all” to “extremely” and transformed to 0–100 [37].

### Psychological Distress

#### Depression and anxiety were used as indicators of psychological distress.

**Patient Health Questionnaire, PHQ-9.**

PHQ-9 is a self-administered nine-item questionnaire used to assess depression. Each item is rated on a four-point scale, from 0 (not at all) to 3 (very often). The score range of PHQ-9 is between 0 and 27. Zero indicates minimal depression symptoms, and 27 indicates severe depression [42]. The reliability and validity of PHQ-9 are supported by the literature [42]. Cronbach’s alpha of PHQ-9 is 0.89, and the construct validity (correlation for PHQ-9 and mental health scale of SF-20) is 0.73.

#### Hospital Anxiety and Depression Scale (HADS)

HADS is a self-administered questionnaire used to screen for depression and anxiety in non-psychiatric clinical populations [43]. It includes fourteen items and two subscales: depression and anxiety. There are seven-item an each subscale, each item is rated on a four-point scale. The score range of each subscale is between 0 and 21 [44]. Zero indicates no case of depression or anxiety, and 21 indicates a severe case of depression or anxiety [44]. The reliability and validity of HADS are supported by the literature [45]. Cronbach’s alphas for the depression and anxiety subscales are 0.79 and 0.85, respectively. The correlations for the life satisfaction questionnaire and HADS-depression and anxiety (construct validity) are respectively -0.66 and -0.42.

### Self-efficacy

#### Self-Efficacy Scale.

Self-efficacy can be defined as the confidence and belief of a person in their ability to avoid back pain symptoms and exacerbations to achieve optimal health. [46]. Self-efficacy scale (SES) adapted from the Kate Lorig scale was used in this study to assess self-efficacy [47]. The self-efficacy scale includes 8 items measured on a 10-point scale, where 1 indicates very uncertain and 10 indicates very certain [47]. The reliability and validity of SES are supported by the literature. The test-retest reliability of SES was 0.87, and there was a significant correlation between self-efficacy and health status with perceived ability [47].

#### Functional status.

Functional status was measured by the following indicators: the Oswestry Disability Index, and the physical function, role physical and social function subscales of SF-12.

##### Oswestry Disability Index (ODI)

The ODI is a self-administered tool developed to assess pain-related disability in individuals with LBP. It includes 10 items on the main daily activities; each item is rated on a six-point scale. The score range of the ODI is between 0 and 100. Zero indicates minimal disability, and 100 indicates “bed-bound or exaggerated symptoms” [48]. The reliability and validity of ODI are supported by the literature. Test-retest reliability of the ODI ranges from 0.81–0.99, and convergent validity (correlation for the ODI and Roland-Morris Questionnaire) is 0.82 [48].

##### SF-12: Physical Functioning, Role-Physical, and Social Functioning Subscales

The following SF-12 subscales were used in this study as indicators of functional status: physical functioning, role-physical and social functioning.

The physical functioning subscale includes two items rated on a 3-point scale, “Yes, limited a lot” to “No, not limited at all” [37]. The role-physical subscale includes two items rated on a 5-point scale, “All of the time” to “None of the time”. Social functioning includes one item rated on a 5-point scale, “All of the time” to “None of the time”. The scores of the physical functioning and role-physical subscales were transformed to 0–100, where 0 indicates a worse condition and 100 indicates a better condition.

#### Measurement tools for other explanatory variables.

##### Sociodemographic Variables

Sociodemographic characteristics included patient sex (men/women), age, education (university or college, secondary and primary school), and marital status (never married, married or common law, divorced or separated, and widowed). Each one of these characteristics was assessed by one question.

##### Healthcare Utilization

Healthcare utilization was assessed by the total number of patient’s visits to the physician, physiotherapist, nurse, and psychologist.

### Statistical Analysis

All descriptive and correlation statistical analyses in this study were done using SAS 9.4 [49], and Structural Equation Modeling (SEM) with ten imputations for missing data was conducted using MPLUS 8 [50]. Pearson correlation was used to assess the correlation between predictor variables to determine multicollinearity. P-values of parameter estimates larger than 0.05 support rejecting a hypothesis. All the studied variables in this project were considered continuous, except for GHP, sex, education, and marital status.

A measurement model was specified for each latent construct (Table 1). SEM was used to assess the relationships between health outcomes and personal and environmental factors and the impact of these relationships on GHP. SEM is an alternative approach to regression used to assess the correlation among health outcomes over time. SEM overcomes the limitations of the regression approach; it enables researchers to use more than one outcome at the same time and to estimate both direct and indirect effects of predictors on an outcome. It allows the simultaneous use of a variable as an outcome and a predictor. This makes it possible to more precisely simulate the causal relationships between studied variables in order to understand the overall relationships among them. SEM comprises both path and measurement models, where the measurement models represent latent variables and provide a more accurate adjustment for a measurement error [51,52].

Five steps were conducted to apply the SEM model: 1) specification: the model should be specified based on a theory; 2) identification: the number of observations in the model should be equal to or more than the number of parameters; 3) estimation: applying various techniques of estimation (e.g., WLSMV); paths in SEM indicate the “beta coefficients”; 4) testing and diagnostics: the goodness of fit of the SEM is examined by Comparative Fit Index (CFI) and Root Mean Square Error of Approximation (RMSEA), RMSEA value ≤ 0.05 and CFI values ≥ 0.9 indicate a good fit model [53]. Lastly, 5) re-specification: according to the results of the model fit, we applied adjustments by changing direction, deleting, or adding some paths to the model [54].

A literature review was firstly conducted to develop the proposed theoretical model. The review summarized the theoretical and statistical relationships in order to strengthen and support the model hypotheses. The theoretical base refers to theories and hypotheses that explain the correlation between the studied variables, while the statistical (empirical) base refers to the significance of the statistical correlation between the studied variables. The summary of the literature supporting for these two bases is presented in S1 Table.

**Fig 1:**
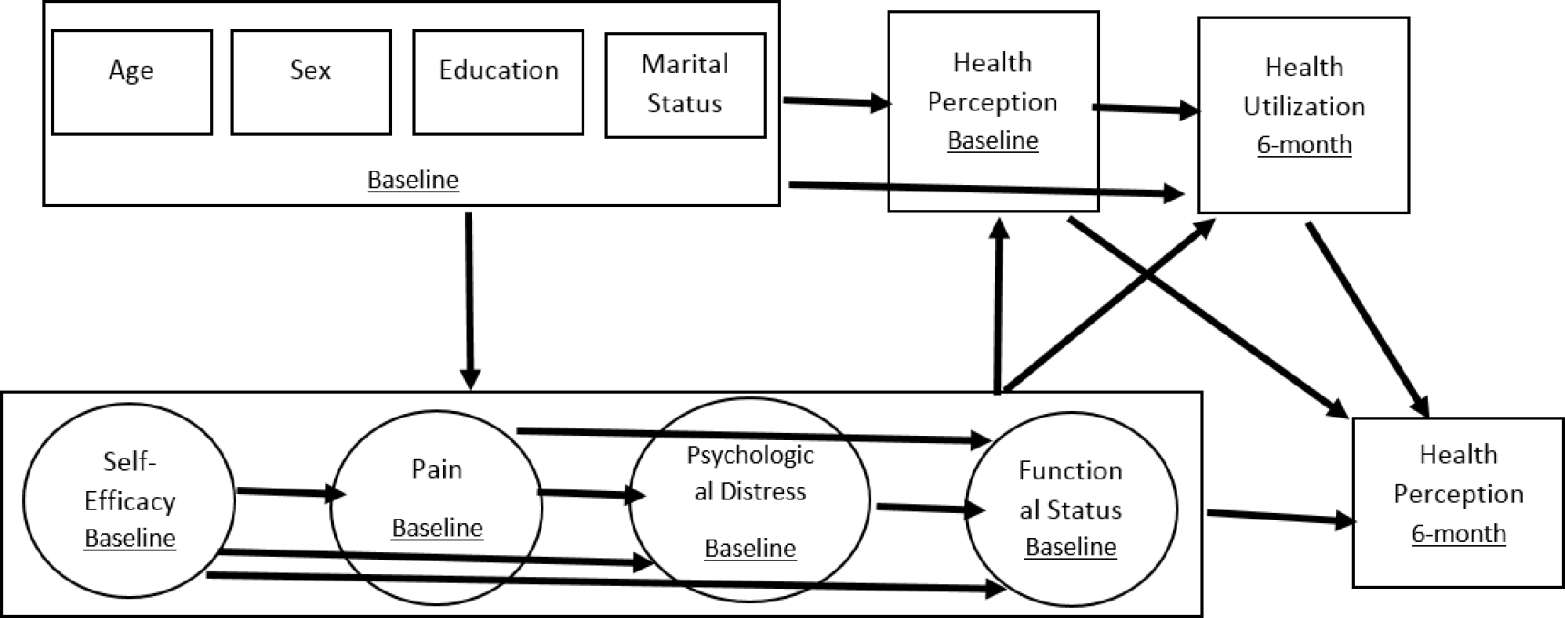
Predictors of health perception model at 6-month - structural paths model. It shows the model of the predictors of health perception overtime, it includes only the structural paths.

The SEM model is provided in Fig 1. For clarity, only structural paths for the studied variables were included in Fig 1. The proposed model includes four latent variables measured at baseline (self-efficacy, pain, psychological distress, and functional ability) and the other seven observed variables (GHP at baseline, GHP at 6-month follow-up, healthcare utilization, sex, age, education, and marital status). For all latent variables and GHP, higher scores indicate better outcomes.

The model specified that self-efficacy was directly affected by age, sex, education, and marital status, while pain was directly affected by age, sex, education, marital status, and self-efficacy. Also, it was hypothesized that psychological stress was directly affected by age, sex, education, marital status, pain, self-efficacy, and functional status. In addition, the model specified that functional status was directly affected by age, sex, education, marital status, pain, self-efficacy, and psychological distress. Lastly, healthcare utilization at 6-month was directly affected by age, sex, education, marital status, pain, psychological symptoms, functional status, and GHP at baseline.

Furthermore, the GHP at baseline was affected directly by age, sex, education, marital status, pain, psychological symptoms, self-efficacy, and functional status at baseline. Lastly, it was hypothesized that the GHP at 6-month was affected directly by age, sex, education, marital status, pain, psychological symptoms, self-efficacy, functional status, GHP at baseline, and healthcare utilization at 6-month. The unit loading identification (ULI) constraint was used for each latent variable.

### Sample Size

Kline recommends that 200 subjects are sufficient to run a structural equation model [55].

## Results

The baseline characteristics of the 314 individuals with LBP are presented in Table 2. The mean age was 62.9 ± 34.57 years, and 60% were women. The participants had moderate pain intensity on the BPI scale (the mean score was 4.75 ± 1.85), mild disability on the ODI (the mean score was 33.1 ± 15.8), and on average within the normal range for depression and anxiety on HADS (6.05 ± 4 and 8.55 ± 3.76, respectively). Only 21 (6.7%) and 37 (11.8%) participants rated their health as “excellent” at baseline and 6-months, respectively.

**Table 2:** Participants’ characteristics at baseline.

Variable	N	Mean (sd)	Missing Data%
Age	314	62.9 (34.57)	6%
BPI-Pain Severity	314	4.75 (1.85)	12%
BPI-Pain Interference	314	4.65 (2.39)	8%
ODI	314	33.1 (15.8)	0.6%
Self-Efficacy	314	6.07 (2.05)	15%
HADS – Depression	314	6.05 (4)	8%
HADS – Anxiety	314	8.55 (3.76)	7%
PHQ-9	314	7.4 (5.85)	8%
SF-12 Bodily Pain	314	35.2 (11.35)	8%
SF-12 Physical Function	314	36.65 (11.6)	4%
SF-12 Role Physical	314	36.25 (9.75)	5%
**SF-12 General Health/ Baseline** Excellent Very Good Good Fair Poor	314	21 (6.7%) 77 (24.4%) 131 (41.7%) 62 (19.7%) 23 (7.5%)	3%
**SF-12 General Health/ 6 months** Excellent Very Good Good Fair Poor	314	37 (11.8%) 94 (30%) 125 (39.7%) 43 (13.7%) 15 (4.8%)	3%
**Changes in SF-12 General Health overtime** No change Improvement Deterioration	314	161 (51.3%) 108 (34.4%) 45 (14.3%)	

BPI: Brief Pain Inventory; HADS: Hospital Anxiety and Depression Scale; ODI: Oswestry Disability Index; PHQ: Patient Health Questionnaire; SF-12: Short Form 12.

### SEM model fit statistics

To achieve an acceptable statistical fit of the model, four error correlations were set between certain indicators that were presented in Table 3. The fit statistics showed that the model was statistically fit (RMSEA = 0.05 and CFI = 0.9). The model explained 37% and 48% of the variance of GHP at baseline and 6-month, respectively.

**Table 3:** The correlation between variable’s errors that was included in the SEM model.

SF12-physical function and SF12-role physical
SF12-social function and ODI
SF12-social function and pain severity of BPI
First and second items of self-efficacy scale

BPI: brief pain inventory; ODI: Oswestry disability index; SF12: Short form 12.

### Factors of self-efficacy

The model showed that aging was the only significant factor that affected self-efficacy (β = 0.13, p-value = 0.03), while sex, education, and marital status did not significantly affect self-efficacy.

### Factors of pain

The model showed that high self-efficacy (β = 0.43, p-value = 0.000), higher educational level (β = 0.18, p-value = 0.006), and being male (β = -0.16, p-value = 0.01) significantly reduced pain intensity. However, age and marital status did not significantly affect the level of pain.

### Factors of psychological distress

The results showed that psychological distress improved with lower pain levels (β = 0.58, p-value = 0.00), and high self-efficacy (β = 0.49, p-value = 0.00). In contrast, age, sex, educational level, and marital status did not significantly affect psychological distress.

### Factors of functional status

The results showed that functional status was significantly affected by pain (β = 0.87, p-value = 0.00), self-efficacy (β = 0.44, p-value = 0.00), psychological distress (β = 0.19, p-value = 0.052), and being male (β = -0.12, p-value = 0.048). On the other hand, age, educational level and marital status did not significantly affect functional status.

### Factors of healthcare utilization at 6-months

The model showed that low pain intensity (β = -0.19, p-value = 0.01) reduced healthcare utilization. Self-efficacy, psychological distress, functional status, health perception, age, sex, educational level, and marital status did not significantly affect healthcare utilization at 6-month.

### Factors of GHP at baseline

The total effect in the SEM model showed that lower pain levels (β = 0.29, p-value = 0.00), lower psychological distress (β = 0.51, p-value = 0.00), higher self-efficacy (β = 0.4, p-value = 0.00), and higher educational level (β = 0.15, p-value = 0.04) significantly improved GHP at baseline. On the other hand, functional status, age, sex, and marital status did not significantly affect GHP at baseline. Table 4 presents the factors of GHP at baseline.

**Table 4:** The effects of the factors of general health perception (GHP) at baseline.

Factor	Total Effect (β) (p-value) [95% Confidence Interval]	Direct Effect (β) (p-value) [95% Confidence Interval]	Indirect Effect (β) (p-value) [95% Confidence Interval]
Pain	0.29 (0.00) [0.18-0.41]	-0.1 (0.6) [-0.42-0.22]	0.39 (0.019) [0.12-0.67]
Self-efficacy	0.4 (0.00) [0.31-0.49]	0.15 (0.02) [0.045-0.26]	0.25 (0.00) [0.18-0.32]
Psychological distress	0.51 (0.00) [0.38-0.65]	0.49 (0.00) [0.36-0.62]	0.02 (0.53) [-0.036-0.08]
Functional status	0.12 (0.51) [-0.18-0.65]	0.12 (0.51) [-0.18-0.42]	–
Gender	0.04 (0.51) [-0.06-0.15]	0.1 (0.054) [0.013-0.19]	-0.06 (0.13) [-0.13-0.01]
Age	0.01 (0.82) [-0.09-0.11]	-0.03 (0.54) [-0.11-0.05]	0.04 (0.22) [-0.01-0.1]
Education	0.15 (0.04) [0.03-0.27]	-0.1 (0.1) [-0.03-0.2]	0.07 (0.1) [0-0.13]
Marital status	0.04 (0.5) [-0.06-0.14]	0.09 (0.11) [-0.09-0.08]	0.05 (0.24) [-0.02-0.11]

### Factors of GHP at 6-month

The total effect in the SEM model showed that some health outcomes significantly improved GHP over time: lower pain levels (β = 0.21, p-value = 0.004), lower psychological distress (β = 0.44, p-value = 0.00), higher self-efficacy (β = 0.36, p-value = 0.00), and GHP at baseline (β = 0.59, p-value = 0.00). On the other hand, functional status, age, sex, education, marital status, and healthcare utilization over the period of 6-months did not significantly affect GHP at 6-month. Table 5 presents the factors of GHP at 6-month.

**Table 5:** The effects of the factors of general health perception (GHP) at 6-month.

Factor	Total Effect (β) (p-value)	Direct Effect (β) (p-value)	Indirect Effect (β) (p-value)
Pain	0.21 (0.004) [0.09-0.33]	-0.03 (0.85) [-0.33-0.26]	0.24 (0.13) [-0.02-0.5]
Self-efficacy	0.36 (0.00) [0.26-0.46]	0.07 (0.28) [-0.04-0.18]	0.29 (0.00) [0.21-0.37]
Psychological distress	0.44 (0.00) [0.29-0.58]	0.13 (0.13) [-0.01-0.27]	0.31 (0.00) [0.21-0.41]
Functional status	0.06 (0.74) [-0.26-0.38]	-0.02 (0.91) [-0.3-0.26]	0.08 (0.46) [-0.1-0.27]
GHP - Baseline	0.59 (0.00) [0.52-0.67]	0.6 (0.00) [0.53-0.67]	-0.006 (0.32) [-0.015-0.005]
Healthcare providers visits	-0.048 (0.22) [-0.11-0.02]	-0.05 (0.22) [-0.11-0.017]	–
Gender	-0.005 (0.94) [-0.11-0.01]	-0.02 (0.77) [-0.11-0.07]	0.01 (0.82) [-0.07-0.09]
Age	-0.002 (0.98) [-0.01-0.09]	-0.03 (0.47) [-0.09-0.035]	0.03 (0.56) [-0.05-0.1]
Education	0.07 (0.27) [-0.04-0.18]	-0.04 (0.51) [-0.13-0.06]	0.11 (0.04) [0.02-0.19]
Marital status	-0.004 (0.95) [-0.11-0.1]	-0.03 (0.56) [-0.13-0.06]	0.03 (0.5) [-0.04-0.1]

## Discussion

To our knowledge, this is the first study to use SEM to assess the impact of pain, self-efficacy, psychosocial distress, functional status, patient characteristics, and healthcare utilization on GHP overtime among people with LBP. Pain, psychological distress, self-efficacy, and GHP at baseline had a significant impact on GHP cross-sectionally and overtime. The results showed that people with low levels of pain had higher levels of GHP. This was supported in the literature, where increasing pain severity decreased GHP among people with LBP [20–22]. Chronic pain may lead to some consequences like physical, psychological, and economic burden [56], which may affect the health status of patients. The model in this study showed that pain affected GHP mainly through psychological distress.

This study also presented that psychological distress at baseline was significantly associated with a low level of GHP at 6-month. The literature shows that people with depression and/or anxiety are more likely to have low GHP [26,27]. Pain, depression, and anxiety may affect activities of daily living, physical health, and mental health, as well as social health [57], which may in turn decrease and deteriorate GHP.

Self-efficacy was shown in this study as a significant predictor of GHP among people with LBP. The model showed that self-efficacy improved GHP through the reduction of psychological distress. Two cross-sectional studies showed that self-efficacy was significantly correlated with GHP among individuals with chronic musculoskeletal pain including osteoarthritis and rheumatoid arthritis [31,32]. According to Social Cognitive Theory [30], self-efficacy promotes and improves GHP by allowing individuals to participate in a healthy lifestyle.

The literature supports that the association between functional status and GHP, tested using multiple regression, is significant among individuals with LBP, where increasing function increases GHP [23, 24, 29]. However, in this study, functional status did not significantly affect GHP at baseline, and 6-months. In part, this is likely related to the difference in the estimation approach used in this study (i.e., regression vs. SEM). In addition, patient characteristics (age, sex, education, and marital status) did not significantly affect GHP cross-sectionally and overtime.

In the proposed model in this study, psychological distress was modeled as a mediator between pain, and self-efficacy and GHP. Lower pain and higher self-efficacy were associated with higher levels of GHP through the reduction of psychological distress. According to Bandura’s study in 1994, self-efficacy is a key factor controlling thought processes and regulating thoughts that produce stress and depression [58]. Also, social cognitive theory and experimental studies support the fact that people who do not believe that they are able to manage threatening events may have high levels of anxiety arousal [59–61]. In addition, the concept of self-efficacy reflects the ability of individuals to avoid the exacerbation of a condition and its comorbidities; by using coping behaviors, self-efficacy may decrease the severity of depression and anxiety [46]. Pain and depression share the same neurological pathway [62]. Normally, response to painful physical stimuli is moderated in the brain by serotonin and norepinephrine. If this mechanism becomes impaired, it affects mood which explains why individuals with LBP generally experienced a state of sadness, hopelessness, or anxiety [63]. In addition, psychological models of pain propose that pain may activate negative emotions including anxiety [64].

Lastly, this study showed changes in the scores of ‘SF-12 General Health’ between the baseline and the 6-month follow-up (Table 2). These changes may reflect the effects of the multidisciplinary intervention received by the participants. This intervention was individualized and applied to all participants, which was controlled in the analysis, reflecting the real life of people with LBP.

### Clinical Implications

This study identified pain intensity, psychological distress, self-efficacy, and GHP at baseline significantly associated with GHP 6 months later among individuals with LBP. Therefore, it is important to assess these health factors to define people with high risk of having low GHP. This can be done by assessing people with LBP using patient-reported outcome measures (PROMs) at the initial visit to refer them to the appropriate treatment. PROMs can be used in clinics for different purposes: to screen for problems, monitor progress over time, and facilitate patient-centred care [65–67]. Administering the PROMs before the clinical visit may reduce the time needed for it by helping clinicians focus on areas most relevant for a given patient; studies have shown that areas flagged as below normal limits are more likely to be discussed in the consultation [65].

Identifying any issues with pain intensity, psychological distress, and/or self-efficacy may contribute to optimizing functional ability, GHP, and QoL to prevent deterioration of patients’ health, enhance self-management approaches, and decrease the cost of health care to society. Also, doing so may contribute to improving healthcare services for individuals with LBP. These contributions will be derived from a better understanding of LBP progression, personalised interventions to individuals with LBP needs, and emphasizing the need for interdisciplinary intervention. The results of this study support the importance of including in LBP programs interventions that improve individuals’ confidence to manage their symptoms and their impact on function. The multidisciplinary/interdisciplinary intervention for LBP is recommended by guidelines for evidence-based primary care management, this interdisciplinary intervention includes physicians, nurses, physical therapists, occupational therapists, psychologists, chiropractors, and pharmacists [68]. The literature supports the effect of interdisciplinary and multidisciplinary treatment among people with LBP; it improves pain, depression, physical and social function, mental health, kinesiophobia, general health, and return to work [69–71]. In addition, cognitive-behavioral therapy (CBT) and self-management has been shown to be an effective treatment to improve self-efficacy among people with LBP [72,73]. The literature shows that high self-efficacy is a significant indicator of return to work among people with LBP [74,75].

Lastly, healthcare providers can use the results of this study to target interventions to people who are more likely to have poor health and focus on improving the significant predictors of GHP. Addressing issues related to pain, psychological distress, and self-efficacy may improve individuals’ GHP. Different treatment procedures can be used to address those issues, such as self-management [76,77], cognitive behavioural therapy [73], and multidisciplinary interventions.

### Limitation

Ideally, all factors within the Wilson and Cleary Model would have been included in the SEM. In this study, we were limited to the variables collected within the CLBP primary project. Some predictors were not covered in the current study, like fatigue and sleep impairment. The findings of this study can be generalized only to people with LBP who receive health care from primary care centers, but not to those who receive care from specialty care. Lastly, the study did not perform sex- or gender-based analyses and age-stratified analyses since the sample sizes for each category were small. It is possible that there are variations in health outcomes and perceptions that are influenced by age-specific pathophysiological factors that this study did not capture.

## Conclusions

GHP at 6-month among people with LBP was found to be associated with pain, psychological distress, self-efficacy, and GHP at baseline. Programs that measure and target these factors as part of the intervention plan can increase individuals’ GHP over time, thereby increasing the likelihood of a successful return to work and increasing social participation. A holistic approach for managing chronic pain is needed to incorporate self-management strategies that can improve self-efficacy, which in turn can help address psychological distress and assist individuals in adopting strategies to reduce the interference of pain on function.

## Supporting information

S1 Table The summary of the correlations between studied variables and the proposed hypotheses. It summarizes the theoretical and statistical association between the studied variables and the proposed hypotheses.
(DOCX)

S2 Table The effects of the factors of the outcomes included in the SEM model. It shows the effects of the factors of the outcomes included in the SEM model.
(DOCX)

STROBE Statement Checklist of items that should be included in reports of *cohort studies* (DOCX)
