# Supplementary material for: Determinants of general health perception among individuals with chronic low back pain overtime: structural equation modeling
Source: PLoS One. 2025 May 23;20(5):e0324101. doi: 10.1371/journal.pone.0324101 (PMC12101730; doi:10.1371/journal.pone.0324101)
Supplement: S1 Table — It summarizes the theoretical and statistical association between the studied variables and the proposed hypotheses. (DOCX) [file pone.0324101.s001.docx]

**S1 Table: The summary of the correlations between studied variables and the proposed hypotheses**

| Health outcomes correlation | Theoretical Base | Statistical Base | Hypothesis |
| --- | --- | --- | --- |
| Health Perceptions and other Health Outcomes | Health perception can be theoretically affected by pain severity , psychological symptom, self-efficacy, and functional status [1, 2] | Health perception can be statistically affected by pain, psychological symptom, self-efficacy, and functional status | - More pain severity and worse psychological symptom will have a significant negative effect on health perception [3-7]  - Higher functional status and self-efficacy will have a significant positive effect on health perception [8-12] |
| Pain and Psychological Symptom | - More pain severity may deteriorate psychological symptom [13]  - Worse psychological symptom may increase pain severity [14, 15] | - More pain severity may deteriorate psychological symptom when the latter is the outcome  - Worse psychological symptom may increase pain severity when the latter is the outcome | - More pain severity will have a significant negative effect on psychological symptom (i.e. increase the severity of psychological symptom). [16-18]  - Worse psychological symptom will have a significant negative effect on pain severity (increase pain severity). [19, 20] |
| Pain and Functional Status | - Pain may deteriorate functional status [21, 22] | - Pain may deteriorate functional status | - More pain severity will have a significant negative effect on functional status (i.e. deteriorate functional status) [15, 23, 24] |
| Psychological Symptom and Functional Status | - Worse psychological symptom may deteriorate functional status [25] | - Worse psychological symptom may deteriorate functional status | - Worse psychological symptom will have a significant negative effect on functional status (i.e. deteriorate functional status) [15, 26] |
| Self-efficacy and Pain | - Higher self-efficacy may decrease pain severity [27, 28] | - Higher self-efficacy may decrease pain severity when the latter is the outcome  - More pain severity may decrease self-efficacy when the latter is the outcome | -Higher self-efficacy will have a significant positive effect on pain severity (i.e. decrease pain severity) [29] |
| Self-efficacy and Psychological Symptom | - Higher self-efficacy may improve psychological symptom [30, 31] | Higher self-efficacy may improve psychological symptom | - Higher self-efficacy will have a significant positive effect on psychological symptom (i.e. decrease the severity of psychological symptom) [32] |
| Self-efficacy and Functional Status | - Higher self-efficacy may improve functional status [33] | - Higher self-efficacy may improve functional status | - Higher self-efficacy will have a significant positive effect on functional status (i.e. improve functional status) [34] |
| Adherence to Treatment and Health Outcomes |  | - Adherence to treatment may decrease pain severity and increase functional status.  - Adherence to treatment can be affected by self-efficacy and psychological symptom | - Adherence to treatment may be negatively affected by depression and positively by self-efficacy.  - Adherence to treatment may positively affect pain severity (decrease) and functional status (improve). |

1. Wilson IB, Cleary PD. Linking clinical variables with health-related quality of life. A conceptual model of patient outcomes. JAMA : the journal of the American Medical Association. 1995;273(1):59-65. Epub 1995/01/04. PubMed PMID: 7996652.

2. Atkins R. Self-efficacy and the promotion of health for depressed single mothers. Mental health in family medicine. 2010;7(3):155-68. Epub 2010/09/01. PubMed PMID: 22477937; PubMed Central PMCID: PMCPmc3018952.

3. Bentsen SB, Wahl AK, Strand LI, Hanestad BR. Relationships between demographic, clinical and pain variables and health-related quality of life in patients with chronic low back pain treated with instrumented fusion. Scandinavian journal of caring sciences. 2007;21(1):134-43. Epub 2007/04/13. doi: 10.1111/j.1471-6712.2007.00440.x. PubMed PMID: 17428225.

4. klemenc-ketiš Z. Predictors of health-related quality of life and disability in patients with chronic non-specific low back pain. Zdrav Vestn 2011;80:379–85.

5. Lame IE, Peters ML, Vlaeyen JW, Kleef M, Patijn J. Quality of life in chronic pain is more associated with beliefs about pain, than with pain intensity. European journal of pain (London, England). 2005;9(1):15-24. Epub 2005/01/05. doi: 10.1016/j.ejpain.2004.02.006. PubMed PMID: 15629870.

6. Bair MJ, Wu J, Damush TM, Sutherland JM, Kroenke K. Association of depression and anxiety alone and in combination with chronic musculoskeletal pain in primary care patients. Psychosomatic medicine. 2008;70(8):890-7. Epub 2008/09/19. doi: 10.1097/PSY.0b013e318185c510. PubMed PMID: 18799425; PubMed Central PMCID: PMCPMC2902727.

7. Antunes RS, de Macedo BG, Amaral Tda S, Gomes Hde A, Pereira LS, Rocha FL. Pain, kinesiophobia and quality of life in chronic low back pain and depression. Acta ortopedica brasileira. 2013;21(1):27-9. Epub 2014/01/24. doi: 10.1590/s1413-78522013000100005. PubMed PMID: 24453639; PubMed Central PMCID: PMCPmc3862015.

8. Borsbo B, Gerdle B, Peolsson M. Impact of the interaction between self-efficacy, symptoms and catastrophising on disability, quality of life and health in with chronic pain patients. Disability and rehabilitation. 2010;32(17):1387-96. Epub 2010/06/02. doi: 10.3109/09638280903419269. PubMed PMID: 20513205.

9. Cross MJ, March LM, Lapsley HM, Byrne E, Brooks PM. Patient self-efficacy and health locus of control: relationships with health status and arthritis-related expenditure. Rheumatology. 2006;45(1):92-6. doi: 10.1093/rheumatology/kei114.

10. Licciardone JC, Gatchel RJ, Kearns CM, Minotti DE. Depression, somatization, and somatic dysfunction in patients with nonspecific chronic low back pain: results from the OSTEOPATHIC Trial. The Journal of the American Osteopathic Association. 2012;112(12):783-91. Epub 2012/12/06. PubMed PMID: 23212429.

11. Takeyachi Y, Konno S, Otani K, Yamauchi K, Takahashi I, Suzukamo Y, et al. Correlation of low back pain with functional status, general health perception, social participation, subjective happiness, and patient satisfaction. Spine. 2003;28(13):1461-6; discussion 7. Epub 2003/07/03. doi: 10.1097/01.brs.0000067091.88283.b6. PubMed PMID: 12838106.

12. Horng Y-S, Hwang Y-H, Wu H-C, Liang H-W, MHE YJ, Twu F-C, et al. Predicting Health-Related Quality of Life in Patients With Low Back Pain. Spine. 2005;30(5):551-5 10.1097/01.brs.0000154623.20778.f0.

13. Sheng J, Liu S, Wang Y, Cui R, Zhang X. The Link between Depression and Chronic Pain: Neural Mechanisms in the Brain. Neural plasticity. 2017;2017:9724371. Epub 2017/07/15. doi: 10.1155/2017/9724371. PubMed PMID: 28706741; PubMed Central PMCID: PMCPMC5494581.

14. National Institute of Mental Health. Chronic Illness and Mental Health: Recognizing and Treating Depression: National Institutes of Health; 2021 [cited 2024 January 19]. Available from: <https://www.nimh.nih.gov/health/publications/chronic-illness-mental-health>.

15. Melton BL, Moqbel M, Kanaan S, Sharma NK. Structural Equation Model of Disability in Low Back Pain. Spine. 2016;41(20):1621-7. Epub 2016/10/19. doi: 10.1097/brs.0000000000001563. PubMed PMID: 26977848; PubMed Central PMCID: PMCPMC5063655.

16. Fernandez M, Colodro-Conde L, Hartvigsen J, Ferreira ML, Refshauge KM, Pinheiro MB, et al. Chronic low back pain and the risk of depression or anxiety symptoms: insights from a longitudinal twin study. The Spine Journal. 2017;17(7):905-12. doi: <https://doi.org/10.1016/j.spinee.2017.02.009>.

17. Ogliari G, Ryg J, Andersen-Ranberg K, Scheel-Hincke LL, Collins JT, Cowley A, et al. Association between pain intensity and depressive symptoms in community-dwelling adults: longitudinal findings from the Survey of Health, Ageing and Retirement in Europe (SHARE). European Geriatric Medicine. 2023;14(5):1111-24. doi: 10.1007/s41999-023-00835-5.

18. Hu Y, Yang Z, Li Y, Xu Y, Tian M, Jiang N, et al. Prevalence and Associated Factors of Depressive Symptoms Among Patients With Chronic Low Back Pain: A Cross-Sectional Study. Frontiers in psychiatry. 2021;12:820782. Epub 2022/02/01. doi: 10.3389/fpsyt.2021.820782. PubMed PMID: 35095623; PubMed Central PMCID: PMCPMC8793741.

19. Tucer B, Yalcin BM, Ozturk A, Mazicioglu MM, Yilmaz Y, Kaya M. Risk factors for low back pain and its relation with pain related disability and depression in a Turkish sample. Turkish neurosurgery. 2009;19(4):327-32. Epub 2009/10/23. PubMed PMID: 19847750.

20. Mok LC, Lee IF. Anxiety, depression and pain intensity in patients with low back pain who are admitted to acute care hospitals. Journal of clinical nursing. 2008;17(11):1471-80. Epub 2008/02/27. doi: 10.1111/j.1365-2702.2007.02037.x. PubMed PMID: 18298508.

21. Zale EL, Ditre JW. Pain-Related Fear, Disability, and the Fear-Avoidance Model of Chronic Pain. Current opinion in psychology. 2015;5:24-30. Epub 2015/04/07. doi: 10.1016/j.copsyc.2015.03.014. PubMed PMID: 25844393; PubMed Central PMCID: PMCPMC4383173.

22. Linton SJ, Shaw WS. Impact of Psychological Factors in the Experience of Pain. Physical therapy. 2011;91(5):700-11. doi: 10.2522/ptj.20100330 %J Physical Therapy.

23. Hall AM, Kamper SJ, Maher CG, Latimer J, Ferreira ML, Nicholas MK. Symptoms of depression and stress mediate the effect of pain on disability. Pain. 2011;152(5):1044-51. Epub 2011/02/11. doi: 10.1016/j.pain.2011.01.014. PubMed PMID: 21306826.

24. Costa Lda C, Maher CG, McAuley JH, Hancock MJ, Smeets RJ. Self-efficacy is more important than fear of movement in mediating the relationship between pain and disability in chronic low back pain. European journal of pain (London, England). 2011;15(2):213-9. Epub 2010/07/27. doi: 10.1016/j.ejpain.2010.06.014. PubMed PMID: 20655254.

25. Linton SJ, Shaw WS. Impact of psychological factors in the experience of pain. Phys Ther. 2011;91(5):700-11. Epub 2011/04/01. doi: 10.2522/ptj.20100330. PubMed PMID: 21451097.

26. Wong JJ, Tricco AC, Côté P, Liang CY, Lewis JA, Bouck Z, et al. Association Between Depressive Symptoms or Depression and Health Outcomes for Low Back Pain: a Systematic Review and Meta-analysis. Journal of general internal medicine. 2022;37(5):1233-46. Epub 2021/08/13. doi: 10.1007/s11606-021-07079-8. PubMed PMID: 34383230; PubMed Central PMCID: PMCPMC8971223.

27. Bandura A, O'Leary A, Taylor CB, Gauthier J, Gossard D. Perceived self-efficacy and pain control: opioid and nonopioid mechanisms. Journal of personality and social psychology. 1987;53(3):563-71. Epub 1987/09/01. PubMed PMID: 2821217.

28. Bandura A. Self-efficacy mechanism in physiological activation and health-promoting behavior. New York: Raven; 1991. 229-70 p.

29. Jackson T, Wang Y, Wang Y, Fan H. Self-Efficacy and Chronic Pain Outcomes: A Meta-Analytic Review. The Journal of Pain. 2014;15(8):800-14. doi: <https://doi.org/10.1016/j.jpain.2014.05.002>.

30. Bandura A. Self-efficacy. In V. S. Ramachaudran (Ed.), Encyclopedia of human behavior. New York: Academic Press; 1994.

31. Alberta Bandura. Social foundations of thought andaction: A socialcognitive theory: Prentice-Hall; 1986.

32. de Moraes Vieira EB, de Goes Salvetti M, Damiani LP, de Mattos Pimenta CA. Self-efficacy and fear avoidance beliefs in chronic low back pain patients: coexistence and associated factors. Pain management nursing : official journal of the American Society of Pain Management Nurses. 2014;15(3):593-602. Epub 2013/07/31. doi: 10.1016/j.pmn.2013.04.004. PubMed PMID: 23891180.

33. Bandura A. Self-efﬁcacy: the exercise of control. New York: WH Freeman and Co.; 1997.

34. van Hooff ML, Vriezekolk JE, Kroeze RJ, O’Dowd JK, van Limbeek J, Spruit M. Targeting self-efficacy more important than dysfunctional behavioral cognitions in patients with longstanding chronic low back pain; a longitudinal study. BMC Musculoskeletal Disorders. 2021;22(1):824. doi: 10.1186/s12891-021-04637-3.
