# Supplementary material for: Determinants of general health perception among individuals with chronic low back pain overtime: structural equation modeling
Source: PLoS One. 2025 May 23;20(5):e0324101. doi: 10.1371/journal.pone.0324101 (PMC12101730; doi:10.1371/journal.pone.0324101)
Supplement: S2 Table — It shows the effects of the factors of the outcomes included in the SEM model. (DOCX) [file pone.0324101.s002.docx]

**S2 Table: The effects of the factors of the outcomes included in the SEM model**

| **Factor** | **Total** | **P-value** | **Direct** | **P-value** | **Indirect** | **P-value** |
| --- | --- | --- | --- | --- | --- | --- |
|  | **Visiting Medical Doctors** | | | | | |
| Pain | -0.19 | 0.01 | 0.01 | 0.95 | -0.21 | 0.24 |
| Self-efficacy | -0.10 | 0.21 | -0.01 | 0.92 | -0.09 | 0.02 |
| Psychological distress | -0.07 | 0.40 | -0.10 | 0.36 | 0.02 | 0.71 |
| Functional status | -0.20 | 0.35 | -0.21 | 0.32 | 0.02 | 0.56 |
| GHP - Baseline | 0.12 | 0.11 | 0.12 | 0.11 | - | - |
| Gender | 0.10 | 0.10 | 0.06 | 0.33 | 0.04 | 0.07 |
| Age | -0.13 | 0.09 | -0.11 | 0.12 | -0.02 | 0.37 |
| Education | -0.12 | 0.06 | -0.10 | 0.10 | -0.02 | 0.51 |
| Marital status | 0.08 | 0.13 | 0.09 | 0.11 | -0.01 | 0.72 |
|  | **Functional Status** | | | | | |
| Pain | 0.87 | 0.00 | 0.76 | 0.00 | 0.11 | 0.00 |
| Self-efficacy | 0.44 | 0.00 | 0.02 | 0.64 | 0.42 | 0.00 |
| Psychological distress | 0.19 | 0.01 | 0.19 | 0.01 | 0.00 | 1.00 |
| Gender | -0.12 | 0.05 | 0.03 | 0.59 | -0.15 | 0.01 |
| Age | 0.06 | 0.31 | 0.01 | 0.89 | 0.06 | 0.33 |
| Education | 0.12 | 0.05 | -0.05 | 0.25 | 0.16 | 0.01 |
| Marital status | 0.02 | 0.72 | -0.05 | 0.28 | 0.07 | 0.24 |
|  | **Psychological distress** | | | | | |
| Pain | 0.58 | 0 | 0.58 | 0.00 | - | - |
| Self-efficacy | 0.49 | 0 | 0.24 | 0.00 | 0.25 | 0 |
| Gender | -0.12 | 0.062 | -0.01 | 0.85 | -0.11 | 0.023 |
| Age | 0.04 | 0.443 | -0.02 | 0.63 | 0.07 | 0.142 |
| Education | 0.12 | 0.074 | -0.01 | 0.79 | 0.13 | 0.004 |
| Marital status | 0.12 | 0.056 | 0.09 | 0.08 | 0.03 | 0.549 |
|  | **Pain** | | | | | |
| Efficacy | - | - | 0.43 | 0 | - | - |
| Gender | -0.164 | 0.01 | -0.15 | 0.015 | -0.017 | 0.554 |
| Age | 0.06 | 0.376 | 0.00 | 0.954 | 0.056 | 0.043 |
| Education | 0.183 | 0.006 | 0.14 | 0.04 | 0.041 | 0.185 |
| Marital status | 0.067 | 0.342 | 0.09 | 0.255 | -0.019 | 0.509 |
|  | **Self-Efficacy** | | | | | |
| Gender | -0.039 | 0.559 | -0.039 | 0.559 | - | - |
| Age | 0.131 | 0.03 | 0.131 | 0.03 | - | - |
| Education | 0.094 | 0.17 | 0.094 | 0.17 | - | - |
| Marital status | -0.045 | 0.509 | -0.045 | 0.509 | - | - |
